# Performance evaluation of eight rapid tests to detect HIV infection: A comparative study from Brazil

**DOI:** 10.1371/journal.pone.0237438

**Published:** 2020-08-13

**Authors:** Feliciana Lage de Oliveira Marinho, Nelson Luiz de Linon Santos, Suzane Pretti Figueiredo Neves, Leonardo de Souza Vasconcellos

**Affiliations:** 1 Department of Complementary Propaedeutic & Post-Graduate Program in Pathology, School of Medicine, Universidade Federal de Minas Gerais, Belo Horizonte, Minas Gerais, Brazil; 2 Instituto Hermes Pardini SA, Vespasiano, Minas Gerais, Brazil; 3 Research Group on Clinical Pathology/Laboratory Medicine, School of Medicine, Universidade Federal de Minas Gerais (GPPCML—CNPq), Belo Horizonte, Minas Gerais, Brazil; Qatar University, QATAR

## Abstract

Rapid tests (RTs), also known as point-of-care tests, usually release results within 30 minutes with no need for a qualified staff, equipment, or laboratory structure. The Brazilian Ministry of Health published a resolution in 2013, recommending the use of RTs for the diagnosis of HIV infection, where one positive RT must be followed by another different RT. This was meant to increase the chance of proper diagnosis in specific settings and special populations. However, data comparing and validating the different HIV RTs available in Brazil are scarce. Therefore, the present study seeks to evaluate eight anti-HIV RTs available in the Brazilian market regarding their analytical performance: sensitivity, specificity, positive and negative predictive values, positive and negative likelihood ratios, and accuracy. We also evaluated the agreement between kits (Kappa index) and the quality of the reading pattern of the tests. This was an observational, analytical, and concordance study, in which previously defined positive and negative samples, based on their serological pattern for anti-HIV antibodies (chemiluminescent immunoassay—ECLIA—used as screening and Western Blot used as the confirmatory test) were tested. Analytical performance and Kappa index were calculated, considering a 95% CI and *p*<0.05. This study identified differences in the performances of the eight tested kits. Six out of eight RTs showed good performance and can be used in the routine laboratory and health care units as screening tests. Regarding the quality of the RT band reading pattern, two brands had several samples showing quite faint bands, thus compromising its use in clinical and laboratory settings.

## Introduction

The use of rapid tests (RTs) for HIV in Brazil dates from 2001 onwards [1], but the first recommendation of the use of RTs for the diagnosis of HIV infection without the need for additional testing was Resolution No. 34 of the Brazilian Ministry of Health of Brazil (BMH), published in 2005 [2]. In 2013, Resolution No. 29 of BMH was published, and a manual entitled “Technical Manual for the Diagnosis of HIV Infection” was released to Brazilian national health services, which currently regulates the diagnosis of HIV infection in Brazil [3–6]. This manual provides a detailed description of the laboratory approaches to characterize HIV infection in flowcharts: a) advocating the use of only RTs for the diagnosis of HIV infection; b) RTs using oral fluid as an alternative diagnostic tool; c) screening immunoassay, either third or fourth generation followed by an HIV viral load in positive samples; or d) screening immunoassay, either third or fourth generation followed by Western Blot/immunoblot (WB/IB) as an alternative.

The use of RTs should preferably be used in situations where there is no laboratory infrastructure or hard-to-reach regions, including Testing and Counseling Centers, Mobile Testing Units, Psychosocial Care Centers, specific and vulnerable population segments, Emergency Care Services, and hospitals. In cases of occupational biological accidents, RTs are recommended for use with pregnant women who have not been tested during prenatal care or whose gestational age does not warrant testing results before delivery, parturient and postpartum women who have not been tested before birth or when the test result is not known at the time of delivery, spontaneous abortion, and people in situations of sexual violence, for prophylaxis purposes. In Brazil, according to current recommendations, one positive test must be followed by another different RT. These tests must be sequential, and it is recommended that, in positive cases, the presence of a virus should be confirmed by the HIV viral load quantification test as soon as possible [3–5].

RTs give same-day results (up to 30 minutes) in a variety of situations and locations [7], and their key features include low cost, quick results, and low degree of complexity of operation and reading [8–10]. Most of these tests are small and portable kits [11]. According to Agustí *et al*. [12] and Louie *et al*. [13], RTs have optimized the medical community’s role in identifying and informing infected individuals, especially in health centers, emergency rooms, doctors' offices, and clinics in general. However, some authors report a low RT sensitivity when compared to the gold standard—a screening test followed by a confirmatory test [14].

There are 41 HIV RT kits that have been registered and approved by the Brazilian Health Surveillance Agency (ANVISA) [15], but few studies comparing their analytical performance and feasibility for routine protocols using only RTs for the diagnosis of the infection are available. Data provided by the BMH report forty scientific references; however, only one nationwide study in Brazil compared the effectiveness of RTs in relation to the gold standard [16]. Therefore, the use of RTs in Brazil, not only for screening, but also for the diagnosis of HIV infection, is still a controversial theme in healthcare routines, and thus warrants further investigation.

This study sought to evaluate eight different RTs used to detect anti-HIV antibodies available in Brazil, regarding the analytical performance (sensitivity, specificity, positive and negative predictive values, positive and negative likelihood ratios, and accuracy), in human blood serum samples with previously defined serological pattern; to evaluate the quality of the reading pattern of the results (bands); and to establish the agreement between a combination of two kits to support current Brazilian recommendations.

## Materials and methods

The Research Ethics Committee (COEP) of Universidade Federal de Minas Gerais (UFMG) approved this research (CAAE 47246115.6.0000.5149) and the need for participant consent was waived.

This is an observational, analytical, and concordance study, which evaluated the analytical performance of eight different commercial RT kits (Table 1) used to detect HIV infection in previously defined serological human serum samples, either positive or negative. The sample calculation test was based on the prevalence of HIV infection in the Brazilian population, considering a sampling error of 0.05% and a significance level of 0.05. Two hundred samples from individuals over 18 months of age were used according to the sample calculation test, and defined as:

- HIV-reactive samples (n = 100)–samples with results above the cut-off value (1.00) in the screening test (electrochemiluminescence, ECLIA-HIV Combi–HIV-1 antigen and total anti-HIV-1 and anti-HIV-2 antibodies—Roche Diagnostics^®^, Mannheim, Germany) and a confirmatory test (Western Blot, New Lav Blot I, BioRad^®^, Marnes la Coquette, France), showing at least two of the following bands: p24, gp41, and gp120/gp160;
- non-reactive HIV samples (n = 100)–samples whose results were below the cut-off value in the screening test (<1.0) and no WB bands.

**Table 1:** List of commercial HIV rapid tests used in this study.

Kit	Manufacturer	Registration (ANVISA)	Sample (type)	Sample volume (μL)	Runtime (minutes)	Methodology	Sensitivity (%)*	Specificity (%)*
Alere Determine HIV 1&2	Alere Medical—Japan	10071770723	Serum, plasma or whole blood	50	20–30	Immunochromatography	99.9	99
DPP Rapid Test HIV 1/2	Labtest—Brazil	10009010335	Serum, plasma, whole blood or oral fluid	10	10–25	Immunochromatography	100	99.9
DS Rapid Test HIV	Labtest—Brazil	10009010324	Serum, plasma or whole blood	5	15–20	Immunochromatography	100	99.7
Imunocrom HIV 1/2	MBiolog Diagnóstico—Brazil	80047580174	Serum, plasma or whole blood	10	10–15	Immunochromatography	98.7	99.9
Imuno-rápido HIV 1&2	Wama Produtos Para Laboratorio Ltda—Brazil	10310030085	Serum, plasma or whole blood	10	10–15	Uninformed	100	99.9
Interkit HIV 1&2	Intertek Katal Biotecnológica—Brazil	10377390219	Serum, plasma or whole blood	20	15–20	Lateral flow immunochromatography	100	99.9
HIV 1/2/O Tri-line	Abon Biopharm (Hangzhou) Co Ltda.—China	10071770815	Serum, plasma or whole blood	25	10–20	Immunochromatography	100	99.8
Bioeasy HIV Test	Standard Diagnostic—South Korea	10071770701	Serum, plasma or whole blood	10	10–20	Uninformed	100	99.8

*According to the manufacturer.

The RTs followed manufacturer instructions. A result was considered valid when the control band appeared in each strip. The reading of the strips was performed by two independent researchers. The samples were initially classified as reactive or nonreactive, according to the presence or absence of a band in the test area, respectively. To compare the quality of the reading pattern of the bands, the following results were classified according to their color intensity: nonreactive (no band seen), “strong” reactive (band equal or stronger to control band), “weak” reactive (weaker than control band, but still easy to see), and “very weak” reactive (a faint band, hard to see).

The following analytical parameters were then established for each kit, with 95% confidence intervals (CI): sensitivity, specificity, positive (PPV) and negative (NPV) predictive values, positive (RL+) and negative (RL-) likelihood ratios, and accuracy. A true positive (TP) result was achieved when it showed reactivity in the screening, WB and RT; true negatives (TN) showed nonreactive results in the screening, WB and RT; false positives (FP) appeared as nonreactive results in screening and WB tests, but reactive in RTs; and false negatives (FN) appeared as nonreactive results in screening, WB tests, and RTs.

Descriptive statistical analysis of categorical variables was performed, and the Kappa test was used to evaluate the agreement between the kits. Good correlation was considered when the kappa index was ≥ 0.85. The Wilcoxon test was applied to compare the values of sensitivity and specificity reported by the manufacturers. The statistical analysis was performed by the IBM SPSS Statistics for Windows, version 19.0 [17], and a 95% CI (p <0.05) was considered significant.

## Results

Of the 200 serum samples analyzed in this study, 115 (57.5%) were male and 85 (42.5%) female. Most of the samples came from patients of the Southeast region of Brazil (50.2%), where the study took place. Distribution according to gender and age demonstrates a higher prevalence of positive results in men aged 26 to 30 years and women aged 31 to 35 years. The average age of the patients was 34.28 (32.75–35.81) years.

Among the eight tested kits, five (Alere Determine HIV 1&2, DPP Rapid Test HIV 1/2, DS Rapid Test HIV, Interkit HIV 1&2, and Bioeasy HIV) achieved 100% sensitivity, while one showed 99% sensitivity (HIV 1/2/O Tri-Line), which was similar to that reported by the manufacturers. However, two kits showed performances far below those reported in prior studies, showing only 92% sensitivity each other (Imuno-rápido HIV 1&2 and Imunocrom HIV 1/2). Many samples showed very weak bands, which were very hard to see, engendering test repetitions to reach a consensus among researchers. In fact, 38 samples showed “weak” reactive bands and 35 showed “very weak” reactive bands, using the Imunocrom HIV 1/2 kit and on Immuno-rápido HIV 1&2 kits. The results were considered as reactive according to the color scale template included within the kit by the manufacturer. According to this, any band color intensity should be considered reactive. This template shows six bands of different color intensity, the first two of which were very difficult to view (similar to the results which were classified as “very weak” reactive by the present work).

These results were considered positive in order to determine the kit’s sensitivity, given that a “band” was seen, although often very faintly (which is different from the true negative result, i.e, no band at all). This will be discussed below.

Regarding the specificity range, there was a variation from 94% to 100%; some of the kits were less specific than those values reported by the manufacturers. The PPV ranged from 90.7% to 100%, while the NPV ranged from 95.5% to 100% (Table 2). Good accuracy was observed in six kits (> 97%).

**Table 2:** Analytical performance of eight rapid tests for detection of HIV infection (95% CI).

KIT	SENSITIVITY	SPECIFICITY	POSITIVE PREDICTIVE VALUE (%)	NEGATIVE PREDICTIVE VALUE (%)	POSITIVE LIKELIHOOD RATIO (LR+)	NEGATIVE LIKELIHOOD RATIO (LR-)	ACCURACY (%)
Alere Determine HIV 1&2	100.0 (-)	94.0 (87.4–97.8)	90.7 (87.4–94.0)	100.0 (-)	0.17 (0.12–0.22)	0.00 (-)	97.0 (94.6–99.4)
DPP Rapid Test HIV 1/2	100.0 (-)	96.0 (90.1–98.9)	93.6 (90.8–96.4)	100.0 (-)	1.00 (-)	0.00 (-)	98.0 (96.1–99.9)
DS Rapid Test HIV	100.0 (-)	99.0 (94.6–100)	98.3 (96.8–99.8)	100.0 (-)	0.00 (-)	0.00 (-)	99.5 (98.5–100)
Imunocrom HIV 1/2	92.0 (84.8–96.5)	100 (-)	100.0 (-)	95.6 (91.6–99.6)	0.00 (-)	8.00 (4.24–11.76)	86.5 (81.8–91.2)
Imuno-rápido HIV 1&2	92.0 (84.8–96.5)	99.0 (94.6–100)	98.2 (96.1–99.5)	95.5 (91.4–99.6)	0.00 (-)	8.08 (4.30–11.86)	87.5 (82.9–92.1)
Interkit HIV 1&2	100.0 (-)	98.0 (93.0–99.8)	96.7 (94.7–98.7)	100.0 (-)	0.50 (0.43–0.57)	0.00 (-)	99.0 (97.6–100)
HIV 1/2/O Tri-line	99.0 (94.6–100)	97.0 (91.5–99.4)	95.1 (90.9–99.3)	99.4 (97.9–100)	0.98 (0.96–1.00)	1.03 (0.00–2.43)	97.5 (95.3–99.7)
Bioeasy HIV Test	100.0 (-)	96.0 (90.1–98.9)	93.6 (90.8–96.4)	100.0 (-)	0.33 (0.27–0.40)	0.00 (-)	98.0 (96.1–99.9)

Samples initially considered discordant from the gold standard results were repeated. When compared to the initial result, no changes were observed after repeating the tests, demonstrating the good reproducibility of the evaluated kits.

Table 3 shows data concerning the agreement (Kappa index) between kits when paired in a combination of two, as two different kits are recommended for a confirmation of an initially reactive sample in Brazil.

**Table 3:** Agreement study—Kappa index (95% CI)—of the eight HIV rapid tests.

	Alere Determine HIV 1 & 2	DPP Rapid Test HIV 1/2	DS Rapid Test HIV	Imunocrom HIV 1/2	Imuno-rápido HIV 1&2	Interkit HIV 1&2	HIV 1/2/O Tri-line
Alere Determine HIV 1&2	-	-	-	-	-	-	-
DPP Rapid Test HIV 1/2	0.73 (0.68–0.78)	-	-	-	-	-	-
DS Rapid Test HIV	0.79 (0.74–0.84)	0.86(0.82–0.90)	-	-	-	-	-
Imunocrom HIV 1/2	0.63 (0.57–0.68)	0.60 (0.55–0.66)	0.67 (0.62–0.72)	-	-	-	-
Imuno-rápido HIV 1&2	0.59 (0.53–0.65)	0.61 (0.56–0.67)	0.66 (0.61–0.71)	0.51 (0.45–0.57)	-	-	-
Interkit HIV 1&2	0.87 (0.83–0. 91)	0.82 (0.78–0.86)	0.86 (0.82–0.90)	0.70 (0.65–0.75)	0.67 (0.62–0.72)	-	-
HIV 1/2/O Tri-line	0.80 (0.75–0.84)	0.73 (0.68–0.78)	0.82 (0.78–0.86)	0.63 (0.57–0.68)	0.58 (0.52–0.64)	0.87 (0.83–0. 91)	-
Bioeasy HIV Test	0.81 (0.77–0.85)	0.73 (0.68–0.78)	0.75 (0.70–0.80)	0.62 (0.57–0.68)	0.57 (0.51–0.63)	0.88 (0.84–0.92)	0.85 (0.81–0.89)

*The value highlighted in red shows the best kappa index.

**Fig 1:**
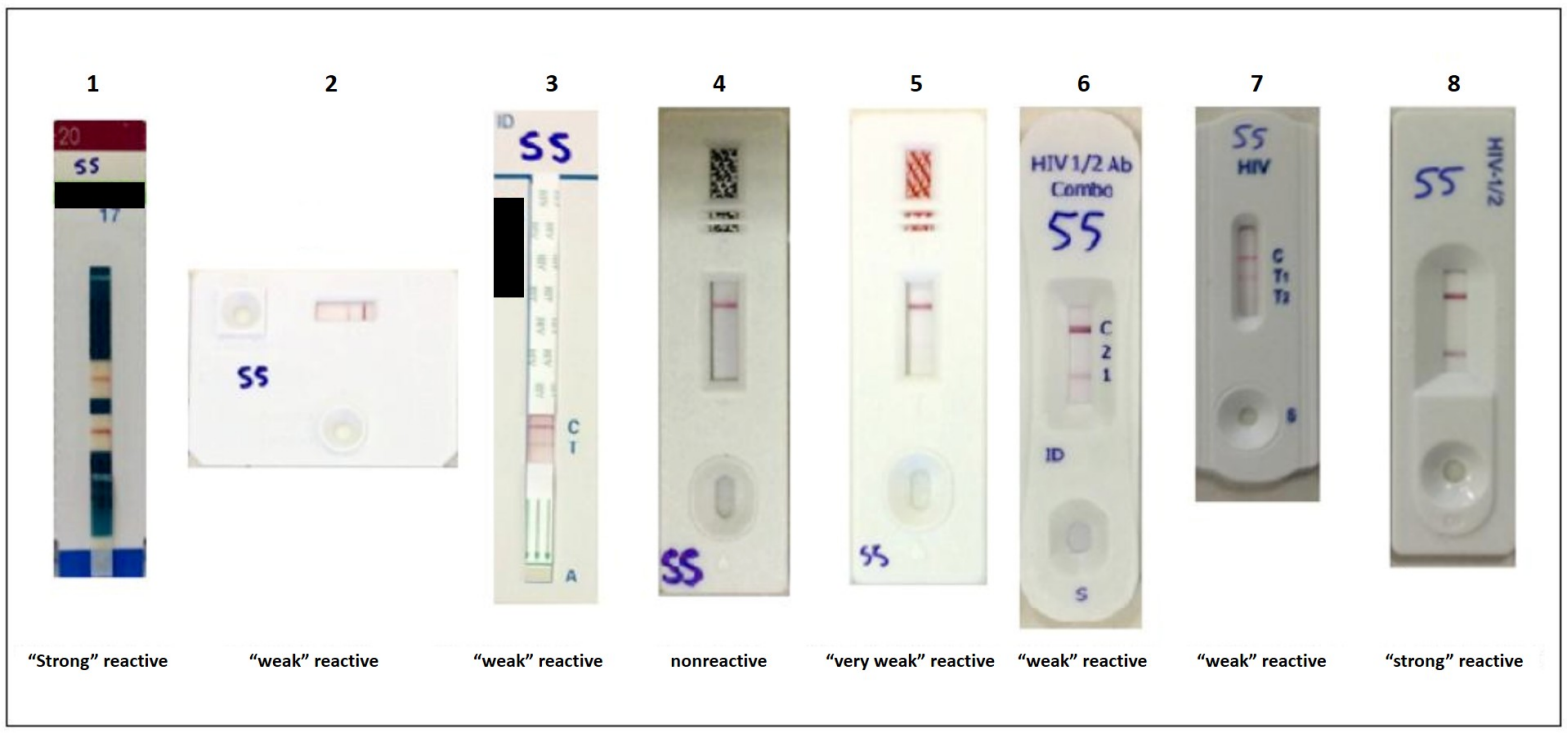
Variation in the results pattern of the same blood sample known as reactive. Different color intensity of the bands is observed in the eight tested devices. 1- Alere Determine HIV 1&2 ("strong" reactive); 2- DPP Rapid Test HIV 1/2 ("weak" reactive); 3- DS Rapid Test HIV ("weak" reactive); 4- Immunocrom HIV 1/2 (nonreactive); 5- Immuno-rápido HIV 1&2 ("very weak" reactive), difficult interpretation due to the very weak intensity of the band; 6- Interkit HIV 1&2 ("weak" reactive); 7- HIV 1/2/O Tri-line ("weak" reactive); 8- Bioeasy HIV Test ("strong" reactive).

Regarding the quality of the reading pattern of the bands, a variation was observed in the visualization pattern of the bands in a similarly known reactive blood sample when different kits were tested (e.g. Fig 1). For these samples, results were classified as “strong”, “weak”, “very weak” reactive and/or non-reactive (S1 Table) and compared with the color intensity guide template of Imuno-rápido HIV 1&2 provided by the manufacturer.

False negative and false positive results are shown in Table 4. The number of true positive results verifies the sensitivity rates.

**Table 4:** True positive and negative, false positive and negative of eight Rapid Test brands to detect HIV infection.

KIT	Manufacturer	TRUE POSITIVE			TRUE NEGATIVE	FALSE POSITIVE	FALSE NEGATIVE
		“strong”	“weak”	“very weak”			
Alere Determine HIV 1&2	Alere Medical–Japan	100	0	0	94	6	0
DPP Rapid Test HIV 1/2	Labtest–Brazil	84	16	0	96	4	0
DS Rapid Test HIV	Labtest–Brazil	99	1	0	99	1	0
Imunocrom HIV 1/2	MBiolog Diagnóstico–Brazil	48	25	19	100	0	8
Imuno-rápido HIV 1&2	Wama Produtos Para Laboratorio Ltda—Brazil	18	58	16	99	1	8
Interkit HIV 1&2	Intertek Katal Biotecnológica–Brazil	96	4	0	98	2	0
HIV 1/2/O Tri-line	Abon Biopharm (Hangzhou) Co Ltda.–China	92	6	1	97	3	1
Bioeasy HIV Test	Standard Diagnostic–South Korea	93	7	0	96	4	0

All tests performed in this study followed the final reading time recommended by each manufacturer, which ranged from 10 to 30 minutes.

## Discussion

The best performing kits based on accuracy analysis were DS Rapid Test HIV and Interkit HIV 1&2. The kits that presented the lowest performance and effectiveness were Imunocrom HIV 1/2 and Immuno-rápido HIV 1&2.

Imunocrom HIV 1/2 showed a large amount of “weak” and “very weak” reactive results (38% and 35%, respectively), as well as Imuno-rápido HIV 1&2 (63% and 17%, respectively). The reading pattern of the bands cannot be mistaken or doubtful to the technical staff because rapid HIV tests provide only qualitative results. Therefore, unnecessary repetitions would be demanded, rising costs in the laboratory. Even with a sensitivity of greater than 90%, we consider its performance inadequate for use in routines and identified the need to use a template with a color scale. Therefore, we suggest that manufacturers conduct a careful revision of these kits, and we would emphasize that clinical laboratories should always proceed with the full verification of the validation processes.

For some authors, the differences found in the sensitivity and specificity when comparing the kits may be due to variations in the ability of the test to detect early seroconversion, due to HIV viral genetic diversity or to the use of different antigens/epitopes or even to the size of the population sample studied [13, 18, 19]. Some studies concluded that RTs were not able to detect antibodies from patients infected with certain HIV subtypes [14]. Machado *et al*. [20] points out that the use of RTs in a specific geographic area should be validated to ensure that the test is adequately sensitive to the circulating HIV types, as positive and negative predictive values may be affected by the relative distribution of the various subtypes in a given region [18, 20].

For Delaney *et al*. [21], HIV RTs show lower sensitivity than do some conventional assays, especially during the early phase of infection. False negative results were also observed in individuals with advanced disease and in some patients on antiretroviral therapy. By contrast, in the present study, all samples had been previously evaluated using the gold standard, a screening test algorithm (ECLIA), followed by a confirmatory test (WB). Therefore, both sensitivity and specificity could be compared to this algorithm.

In Brazil, according to Resolution 29 of BMH [3–6], if only RTs are used in the diagnosis, more precision can be observed when a reactive result is repeatedly confirmed by another RT from another manufacturer. Thus, in the paired evaluation of RTs, six combinations of paired kits achieved Kappa > = 0.85. However, it is prudent to conduct further studies that prove the diagnostic efficiency of these RTs and other RTs available on the market.

Considering the sensitivity criteria for the diagnostic use of RTs proposed by the BMH [3–6] (sensitivity ≥ 99.5%), five out of eight kits were approved in this study. Concerning the specificity (specificity ≥ 99.0% according to the MS), only two met the requirements for routine care. Unfortunately, the technical manual fails to mention the studies that gave origin to this information and how these sensitivity/specificity values have been obtained. Likewise, not all RT use instructions report information in which reference tests were used, nor the description of the tested population.

Ferreira Junior *et al*. [16], in 2005, evaluated seven different RTs commercially available in Brazil and found that of these, only four were considered acceptable for diagnosis. In the present study, even though there were differences in sensitivity, specificity, positive and negative predictive values, positive and negative likelihood ratios, and accuracy, of the eight evaluated RTs, six showed accuracies > 97%. Therefore, it is important for physicians to be aware of such discrepancies and that any conflicting results with epidemiology and/or clinics should be interpreted with caution. In cases of doubt, conventional testing is always recommended (a screening immunoassay, followed by a confirmatory test on those initially reactive samples).

Kosack *et al*. [22], in 2017, evaluated eight HIV RTs using specimens collected from Médecins Sans Frontières sites, between 2011 and 2015, in five African countries. They found a substantial number of false reactive test results evidencing a poor positive predictive values and concluded that only one test met the recommended thresholds for RTs of ≥ 99% sensitivity and ≥ 98% specificity. However, according Johnson *et al*. [23], commenting Kosack *et al*. article, a single reactive test result is never sufficient to make an HIV-positive diagnosis and the countries and programs need to ensure that they are following World Health Organization (WHO) recommended HIV testing strategies and use a validated testing algorithm. In Kosack *et al*. [24] response letter, individual test performances in the target population must be known and the false reactivity of individual samples with multiple tests considered.

Kravitz Del Solar *et al*. [25] recommends that all countries use WHO pre-qualified RTs within the recommended strategies and algorithms for HIV testing. They also support validation of HIV testing algorithms using in-country specimens to determine optimal performance, and the reverification testing of all people diagnosed with HIV prior to starting treatment as an essential quality assurance measure. In our study, the analytical performance of eight different commercial RT kits used to detect HIV infection were evaluated in previously defined serological human serum samples, either positive or negative, according to the guidelines form BMH and WHO recommendations.

There is no evidence in the literature of other studies that evaluated the same RTs tested in our study. Therefore, our results were important to alert the population which RTs had the best diagnostic accuracy when used in sequence, according to the standards of sensitivity and specificity required by BMH.

In conclusion, performance differences were found among the eights tested kits. Six achieved good agreement for use in routines whenever a result is initially reactive; however, the best combination regarding accuracy was the Bioeasy HIV Test and the Interkit HIV 1&2. It is worth noting that every trial, whether rapid or conventional, requires internal validation, as described in good clinical laboratory practices.

## Supporting information

S1 Table (XLSX)
